# Treatment of myofascial trigger points in patients with chronic shoulder pain: a randomized, controlled trial

**DOI:** 10.1186/1741-7015-9-8

**Published:** 2011-01-24

**Authors:** Carel Bron, Arthur de Gast, Jan Dommerholt, Boudewijn Stegenga, Michel Wensing, Rob AB Oostendorp

**Affiliations:** 1 Scientific Institute for Quality of Healthcare, Radboud University Nijmegen Medical Centre, Nijmegen, The Netherlands; 2 Private Practice for Physical Therapy for Neck, Shoulder and Upper Extremity Disorders, Groningen, The Netherlands; 3 Department of Orthopedic Surgery, Clinical Orthopedic Research Center Midden-Nederland, Diakonessenhuis, Utrecht, The Netherlands; 4 Bethesda Physiocare, Bethesda, MD, USA; 5 Department of Oral and Maxillofacial Surgery, University Medical Centre Groningen, University of Groningen, Groningen, The Netherlands

## Abstract

**Background:**

Shoulder pain is a common musculoskeletal problem that is often chronic or recurrent. Myofascial trigger points (MTrPs) cause shoulder pain and are prevalent in patients with shoulder pain. However, few studies have focused on MTrP therapy. The aim of this study was to assess the effectiveness of multimodal treatment of MTrPs in patients with chronic shoulder pain.

**Methods:**

A single-assessor, blinded, randomized, controlled trial was conducted. The intervention group received comprehensive treatment once weekly consisting of manual compression of the MTrPs, manual stretching of the muscles and intermittent cold application with stretching. Patients were instructed to perform muscle-stretching and relaxation exercises at home and received ergonomic recommendations and advice to assume and maintain good posture. The control group remained on the waiting list for 3 months. The Disabilities of Arm, Shoulder and Hand (DASH) questionnaire score (primary outcome), Visual Analogue Scale for Pain (VAS-P), Global Perceived Effect (GPE) scale and the number of muscles with MTrPs were assessed at 6 and 12 weeks in the intervention group and compared with those of a control group.

**Results:**

Compared with the control group, the intervention group showed significant improvement (*P *< 0.05) on the DASH after 12 weeks (mean difference, 7.7; 95% confidence interval (95% CI), 1.2 to 14.2), on the VAS-P1 for current pain (mean difference, 13.8; 95% CI, 2.6 to 25.0), on the VAS-P2 for pain in the past 7 days (mean difference, 10.2; 95% CI, 0.7 to 19.7) and VAS-P3 most severe pain in the past 7 days (mean difference, 13.8; 95% CI, 0.8 to 28.4). After 12 weeks, 55% of the patients in the intervention group reported improvement (from slightly improved to completely recovered) versus 14% in the control group. The mean number of muscles with active MTrPs decreased in the intervention group compared with the control group (mean difference, 2.7; 95% CI, 1.2 to 4.2).

**Conclusions:**

The results of this study show that 12-week comprehensive treatment of MTrPs in shoulder muscles reduces the number of muscles with active MTrPs and is effective in reducing symptoms and improving shoulder function in patients with chronic shoulder pain.

**Trial registration number:**

ISRCTN: ISRCTN75722066

## Background

Shoulder pain is a common musculoskeletal problem. In several countries, the 1-year prevalence is estimated to be 20% to 50% [1,2]. The annual incidence of shoulder pain and symptoms in Dutch primary care practice ranges from 19 to 29.5 per 1,000 [3,4]. Shoulder pain is the main contributor to nontraumatic upper-limb pain, in which chronicity and recurrence of symptoms are common [5,6]. The most common cause of shoulder pain is considered to be subacromial impingement syndrome (SIS), which causes inflammation and degeneration of subacromial bursae and tendons [7,8]. SIS was first described in 1867 by French anatomist and surgeon Jarjavay [9], was reintroduced in 1972 by Neer [10]. Although the interpretation of the physical signs during shoulder examinations is far from reliable [11,12], the diagnosis of SIS is based mainly on the clinical picture of pain in the shoulder as described by Neer [13]. The clinical picture consists of an arc of pain, crepitus and muscle weakness as well as a positive impingement test, which means complete relief of pain with forced forward elevation of the upper arm after injection of a local anesthetic into the subacromial space [11]. Scientific evidence from randomized, controlled trials (RCTs), meta-analyses or systematic reviews of RCTs regarding the effectiveness of multimodal rehabilitation, injection therapy, medication, surgery, physical therapy or the application of other therapies in patients with shoulder pain is conflicting or lacking [14-24], which justifies a search for an alternative explanation of shoulder pain, regardless of whether the patient is diagnosed with SIS.

**Figure 1:**
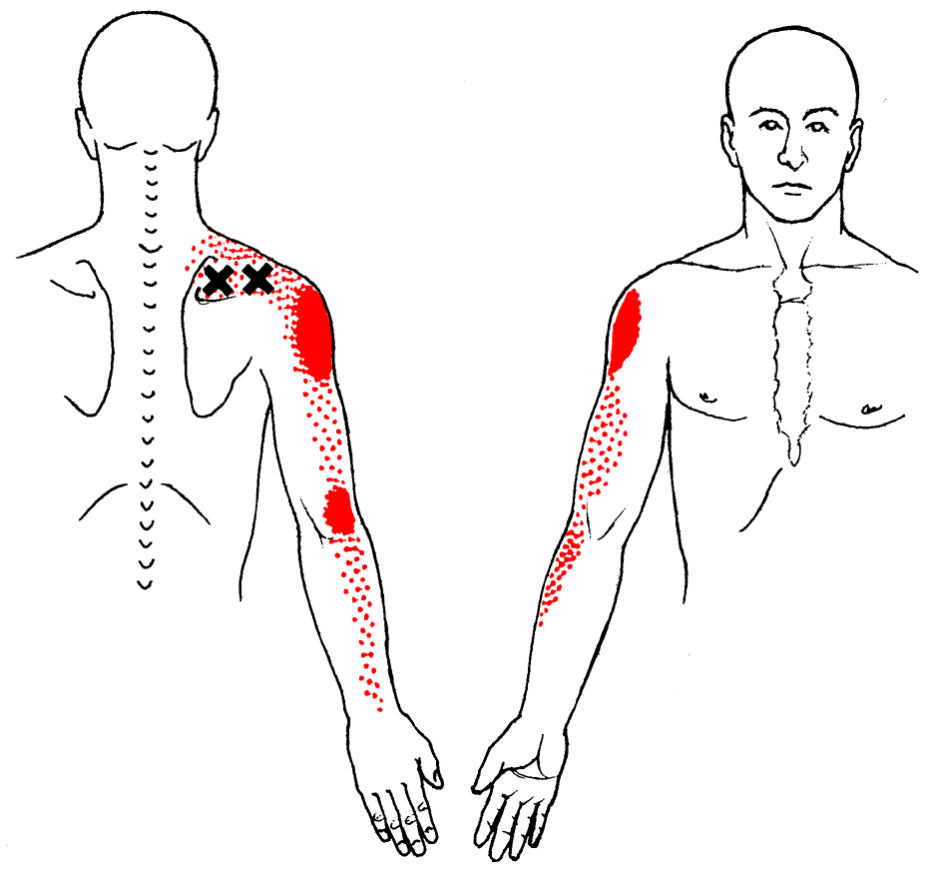
Referred pain pattern (red) from supraspinatus muscle MTrP

**Figure 2:**
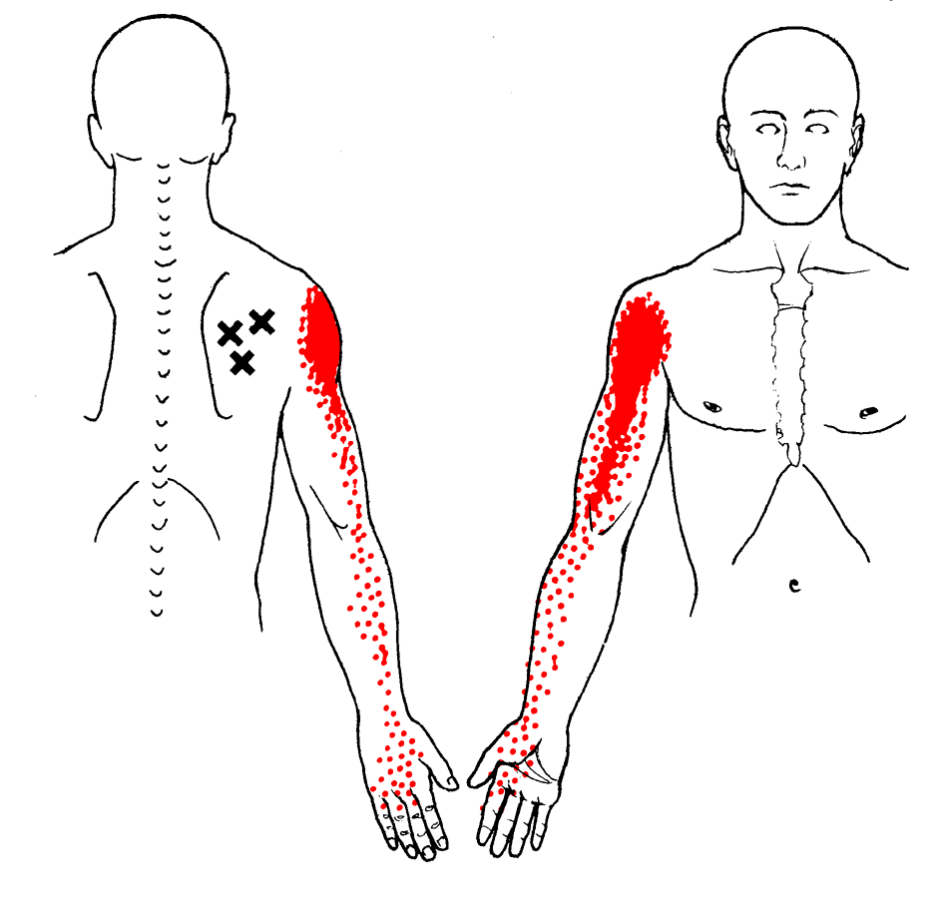
Referred pain pattern (red) from infraspinatus muscle MTrP

**Figure 3:**
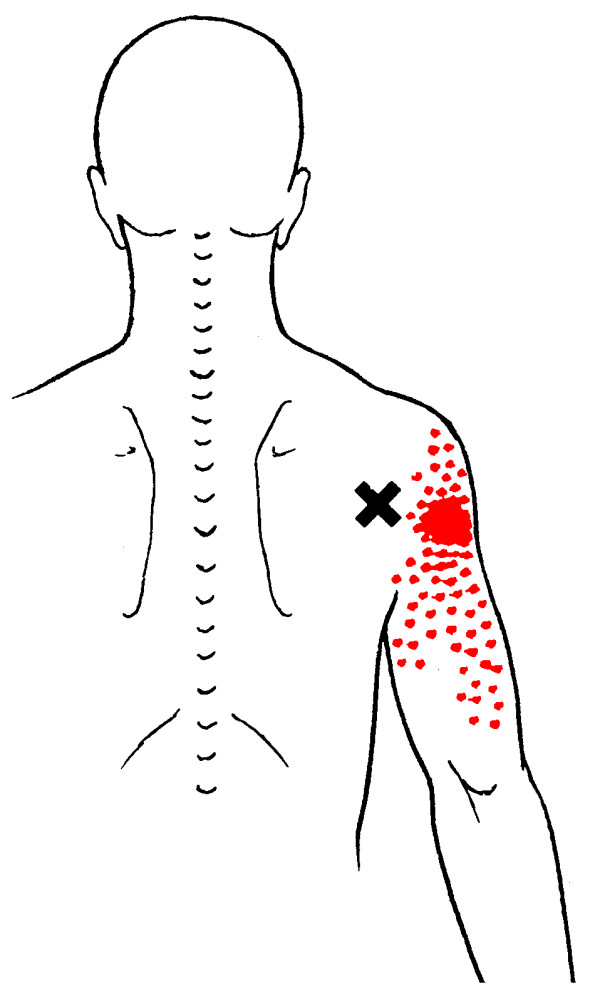
Referred pain pattern from teres minor muscle MTrP

**Figure 4:**
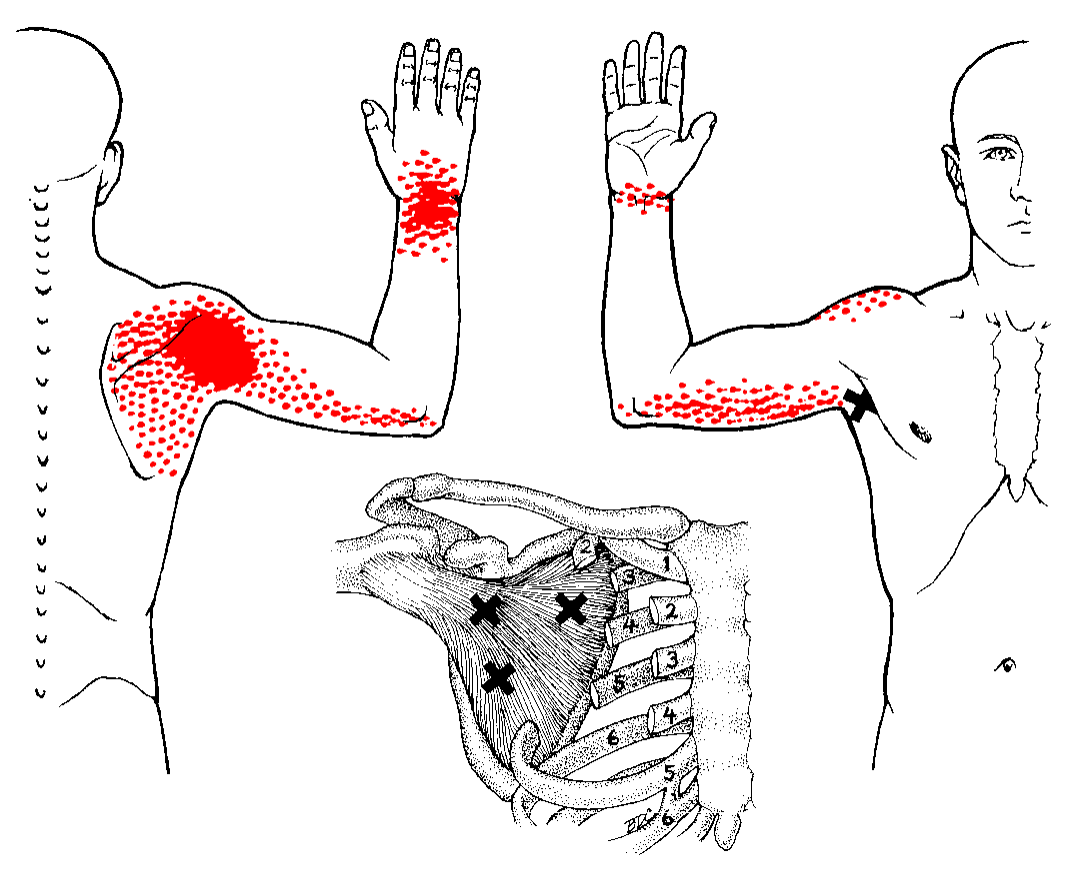
**Referred pain pattern from subscapularis muscle MTrP**. The referred pain patterns according to Simons *et al*. [26]. MTrPs are indicated by *X*. Illustrations courtesy of LifeART/MEDICLIP [88].

A common cause of muscle pain is myofascial pain caused by myofascial trigger points (MTrPs) [[25]; Bron et al, unpublished work]. MTrPs in the shoulder muscles produce symptoms similar to those of other shoulder pain syndromes, including pain at rest and with movement, sleep disturbances and pain provocation during impingement tests [26]. Clinical, histological, biochemical and electrophysiological research has provided biological plausibility for the existence of MTrPs [27-36]. As a result, the role of MTrPs in musculoskeletal pain is increasingly accepted in the medical literature. MTrPs are defined as exquisitely tender spots in discrete taut bands of hardened muscle that produce symptoms known as myofascial pain (Figures 1, 2, 3, 4).

MTrPs are classified into active and latent trigger points. According to Simons *et al*., "An active MTrP causes a clinical pain complaint. It is always tender, prevents full lengthening of the muscle, weakens the muscle, refers a patient-recognized pain on compression, mediates a local twitch response of muscle fibers when adequately stimulated and, when compressed within the patient's pain tolerance, produces referred motor phenomena and often autonomic phenomena, generally in its pain reference zone, and causes tenderness in the pain reference zone" [[26], page 1]. Simons *et al*. defined a latent MTrP as "clinically quiescent with respect to spontaneous pain; it is painful only when palpated. A latent MTrP may have all the other clinical characteristics of an active MTrP and always has a taut band that increases muscle tension and restricts range of motion" [[26], page 4]. Palpation is still considered the only reliable clinical method of diagnosing MTrPs. Previous studies have shown that trained physical therapists can reliably detect MTrPs by palpation [37,38]. Although magnetic resonance elastography and ultrasound imaging studies have shown potential in allowing clinicians to visualize MTrPs, their clinical usefulness has yet to be established [31,32].

Manual techniques, spray and stretch and trigger point needling can inactivate MTrPs. MTrP inactivation may be combined with ergonomic advice, active exercises, postural correction and relaxation if and when appropriate [26,39-45]. Treatment of MTrPs is rarely included in systematic reviews of the effectiveness of conservative interventions in patients with shoulder pain. However, several case studies have suggested that the treatment of MTrPs in patients with shoulder pain may be beneficial, although well-designed controlled studies are still lacking [46-51]. Recently, Hains *et al*. [52] compared ischemic compression of relevant MTrPs (intervention) with ischemic compression of irrelevant MTrPs (sham treatment). The results of this study suggest that ischemic compression of MTrPs in shoulder muscles may reduce the symptoms of patients experiencing chronic shoulder pain.

The aim of the current study was to assess the effectiveness of a comprehensive treatment program of MTrPs in shoulder muscles on symptoms and the functioning of the shoulder in patients with chronic nontraumatic shoulder pain compared with a wait-and-see approach.

## Methods

A single-blinded RCT was conducted, which was approved by the Medical Ethics Committee of the Radboud University Nijmegen Medical Centre, Nijmegen, the Netherlands [CMO 2007/022]. This RCT is registered at Current Controlled Trials [ISRCTN75722066], and the study protocol was published previously [53].

### Patients in the study sample

Between September 2007 and December 2009, all consecutive patients with shoulder pain referred to a primary care practice for physical therapy were potential study participants. The patients were self-referred or were referred by general practitioners, orthopedic surgeons, neurologists or physiatrists. Patients were eligible if they had had unilateral nontraumatic shoulder pain for at least 6 months, were between ages 18 and 65 years and had a clinical presentation that did not warrant referral for further diagnostic screening. Excluded from the study were patients who previously had been diagnosed with shoulder instability; shoulder fractures; systemic diseases such as rheumatoid arthritis, Reiter's syndrome, or diabetes; or whose medical history or physical examination suggested neurological diseases or other severe medical or psychiatric disorders. Patients with signs and symptoms of a primary frozen shoulder were also excluded. Because the questionnaires were in the Dutch language, patients had to understand written and verbal Dutch. The lead investigator (CB) checked all available information from referral letters and additional information from the patients. All eligible patients were invited to participate in the study. The patients were informed of the study before the first assessment and signed a written, informed consent statement.

### Data assessment

Two research assistants (MO and MB; see Acknowledgements), each with 30 years of clinical experience in primary care practice and more than 5 years of experience in identifying and treating MTrPs, performed the physical examination, including the assessment of passive range of motion (PROM) of the shoulder and the MTrP palpation of the shoulder muscles. The total number of shoulder muscles with active and latent MTrPs was counted. The research assistants were blinded to the patient treatment allocations during the entire study period. The assessments were made at intake, prior to randomization and at 6 and 12 weeks. For every patient, only one observer was active. A detailed medical history was completed, which included demographic variables and potential prognostic factors [54,55] and a set of self-administered questionnaires regarding outcome measurements, including the Disabilities of Arm, Shoulder and Hand (DASH) questionnaire, the Visual Analogue Scale for Pain (VAS-P), the RAND Medical Outcomes Study 36-Item Short Form Health Survey (RAND-36) and the Beck Depression Inventory, Second Edition (BDI-II). A third research assistant (IS; see Acknowledgements) transferred the collected data to a worksheet. After the data from the worksheet were transferred into the statistical software packages Systat 12, SigmaPlot 11 and SigmaStat 3.11 for Windows software (Systat Inc., Richmond, CA, USA), the lead investigator (CB), who was blinded to the patients' treatment allocation until all statistical tests were performed, analyzed the data. Blinding of the patients and the treating physical therapists was impossible because of the treatment characteristics.

### Sample size

The planned sample size was determined on the basis of an assumed mean improvement of the primary outcome, a DASH questionnaire score of 15 points (SD ± 22), which implies an effect size of 0.68 [56]. To test the null hypothesis at α = 0.05 with 90% power and assuming a uniform dropout rate of 5%, it was calculated that 52 patients in each group would be required.

### Randomization

After collection of patients' data at baseline, the included patients were randomly assigned to either the intervention group or the wait-and-see group. A research assistant (IS) performed the randomization by generating random numbers using Research Randomizer software (http://www.randomizer.org/) [57]. These numbers were stored on a computer and were accessible only by the assistant. No stratification or blocking strategies were used.

### Interventions

The patients in the intervention group were treated by a physical therapist once weekly for a maximum of 12 weeks. Five physical therapists were involved in the treatment of the patients. All participating physical therapists were experienced in treating patients with persistent shoulder pain and MTrPs. They were trained and skilled in the identification and treatment of MTrPs and had successfully completed a certification-training program in trigger point therapy.

**Figure 5:**
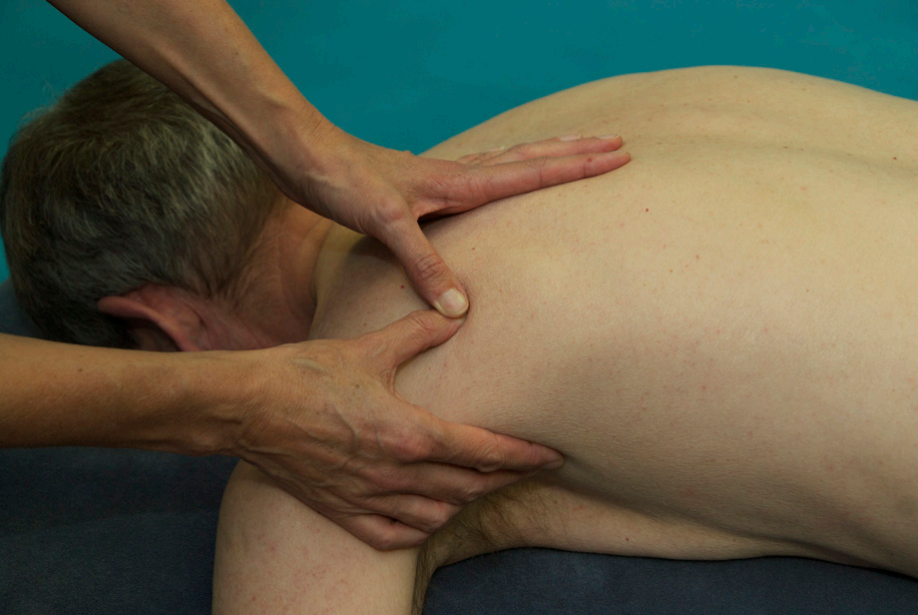
**Manual compression on the MTrP in the infraspinatus muscle of the left shoulder**.

**Figure 6:**
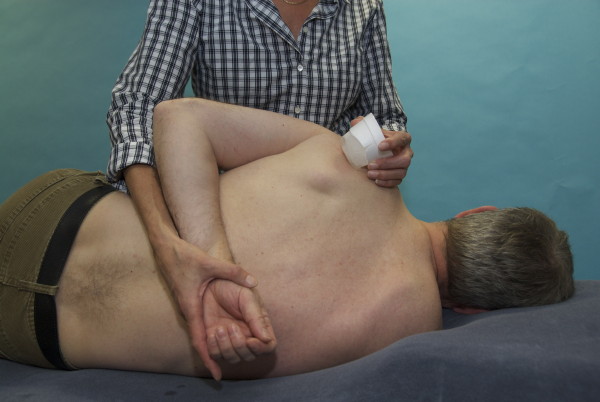
**Stroking with ice (in a polystyrene cup) in unidirectional parallel strokes combined with gentle muscle stretching applied for the infraspinatus muscle of the left shoulder while the patient was lying on one side**.

**Figure 7:**
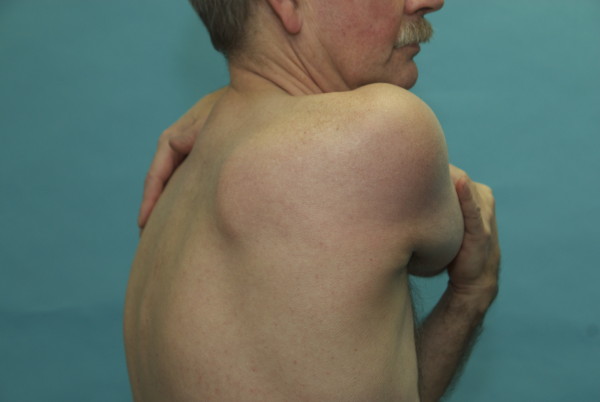
**Cross-body muscle-stretching exercise for posterior shoulder muscles, including the infraspinatus muscle**.

The treatment started with inactivation of active, pain-producing MTrPs by manual compression. The physical therapist applied gentle, gradually increasing pressure on the MTrP until the finger encountered a definite increase in tissue resistance. At that point, the patient commonly would feel a certain degree of discomfort or pain. The pressure was maintained until the therapist sensed relief of tension under the palpating finger or the patient experienced a considerable decline in pain. At that point, the therapist could repeat this procedure several times until pressure on the MTrP would provoke only a little discomfort without pain. This technique was combined with other manual techniques, such as deep stroking (pressure directed along the length of the taut band) or strumming (pressure applied perpendicularly across the muscle fibers). Both techniques can manually stretch the trigger point area and the taut band. These manual techniques could be preceded or followed by "intermittent cold application by using ice-cubes followed by stretching the muscle" according to Simons *et al*. [26]. The effectiveness of muscle-stretching exercises was enhanced by including short isometric contractions and relaxation (hold-relax). Patients were instructed to perform simple gentle static stretching and relaxation exercises at home several times during the day. When appropriate, the relaxation exercises were augmented by using a portable myofeedback device (Myotrac I; Thought Technology, Montréal, QC, Canada). Furthermore, patients were instructed to apply heat, such as a hot shower or hot packs, for muscle relaxation and pain relief at least twice every day. All patients received ergonomic advice and instructions to assume and maintain good posture [58,59]. The content and aim of each session varied on the basis of the specific findings from the initial evaluations and patients' responses to previous treatment sessions. All individual treatments, however, were consistent with the limits of the treatment protocol (Figures 5, 6, 7) [53].

### Stop rule

Treatments were discontinued when patients were completely free of symptoms or when the patient and physical therapist agreed that treatment would not further benefit the patient. Participation in the study continued unless patients decided to stop participation in the study. Patients were free to withdraw from the study at any time without consequences for their treatment.

### Treatment integrity

To enhance the integrity of the interventions, all participating physical therapists were allowed to discuss the content of each therapy session with the lead investigator (CB) without releasing names or any other information that could jeopardize the blinding of the lead investigator. After 6 and 12 weeks of treatment, the lead investigator interviewed the patients of the intervention group to ensure that the received treatments had been consistent with the study protocol.

#### Wait and See

Patients in the control group remained on a waiting list and were informed that they would receive the same physical therapy as the patients in the intervention group after 3 months had passed. They were instructed not to change the self-management of their shoulder pain. If they were using either prescribed or over-the-counter medication, they were encouraged to continue the medication at their own discretion because of their participation in the study. In addition, they were requested to report any other intervention or other relevant change during the study period. Every 6 weeks they visited the physical therapy practice and provided research data similar to the patients from the intervention group. After 12 weeks, they started physical therapy.

### Outcome measures

#### Primary outcome measure

The Disabilities of Arm, Shoulder and Hand (DASH) questionnaire is an internationally widely used multidimensional 30-item self-report measure focusing on physical function, pain and emotional and social parameters [60]. The score ranges from 0 to 100 whereby a higher score indicates greater disability. The Minimal Clinically Important Difference (MCID) is approximately a 10-point difference between pre- and posttreatment [56,61,62]. The DASH questionnaire is a reliable, valid questionnaire and is considered to be one of the best questionnaires for patients with shoulder symptoms [61,63].

### Secondary outcome measures

The Visual Analogue Scale for Pain (VAS-P) is a self-report scale consisting of a horizontal line 100 mm in length that is anchored by the ratings "no pain" at the left side (score 0) and "worst pain imaginable" at the right side (score 100) [64-66]. The VAS-P was used to measure pain at the current moment (VAS-P1), average pain during the past 7 days (VAS-P2) and the most severe pain during the past 7 days (VAS-P3). A 14-mm change is considered to be a MCID in patients with rotator cuff disease [67-70].

To assess Global Perceived Effect (GPE), the subjects rated the effect of treatment on an ordinal 8-point scale with categories ranging from "1 = much worse" to "8 = completely recovered." The GPE score was then dichotomized into the number of patients whose pain had improved (from slightly improved to completely recovered) versus patients whose pain had not improved (from unchanged to much worse). The GPE scale has good test-retest reliability and correlates well with changes in pain and disability [71].

The PROM of the shoulder was measured using a handheld digital inclinometer (Saunders Group Inc., Chaska, MN, USA) and recorded in degrees. Forward elevation of the shoulder, external rotation and cross-body adduction were measured in the supine position, internal rotation in prone position and glenohumeral abduction in the upright position. The range of motion of the nonpainful shoulder was used as a reference. A detailed description of the goniometric measurement of the PROM is published in the report describing the design of this study [53].

The total number of shoulder muscles with MTrPs was counted using the same methods as at baseline and then compared with the baseline measurements. While the patient was in a supine or prone position, depending on the muscle that was examined, 17 muscles (see appendix) were palpated bilaterally for the presence of a taut band, spot tenderness, the presence of a nodule, local twitch response and local and referred pain. When the patient recognized the pain from compression on the tender spot, the MTrPs were considered to be active. When the pain was only local and not familiar, MTrPs were considered to be latent [26,37,53]. At 6 and 12 weeks, participants were asked to complete a self-assessment form, which included questions regarding whether they had changed their self-management or had received any medical treatment that could have influenced their shoulder pain.

### Statistical analysis

Analyses were performed according to the intention-to-treat principle. Both groups were compared for baseline characteristics using a *t*-test and a χ^2 ^test for binominal variables. For the DASH, VAS-P and the number of muscles with MTrPs, the *t*-test for normally distributed data was used to assess the difference between the two groups at week 6 and week 12. We considered a mean difference of more than 10 points on the DASH as a MCID. Effect sizes measured using Cohen's *d *were calculated to examine the average impact of the intervention [72]. According to the method of Cohen, *d *≈ 0.2 indicates small effect and negligible clinical importance, *d *≈ 0.5 indicates medium effect and moderate clinical importance and *d *≈ 0.8 indicates a large effect and crucial clinical importance [73]. To compare patients who improved by more than 10 points with patients who improved by less than 10 points on the DASH questionnaire, we calculated the relative risk (RR) and the 95% confidence interval (95% CI). To examine the impact on individual patients in more detail, we dichotomized participants' measures of GPE into improved versus not improved. The proportions of patients who had clinically improved between groups were compared by calculating the RR and the 95% CI at 6 weeks and 12 weeks. Pearson's correlation coefficients were used to relate the variables of number of muscles with active MTrPs and the DASH questionnaire score.

In addition, the effect of the intervention was evaluated by using regression analysis. Covariates in this multiple linear regression model were the DASH questionnaire score at 12 weeks as the dependent variable, the group variable as the DASH questionnaire score at baseline, and the number of muscles with active MTrPs at intake as the number of muscles with latent MTrPs at intake, as well as the PROM.

To evaluate the success of the blinding procedure, both observers were asked to identify the treatment allocation. A goodness-of-fit χ^2 ^test was used to determine whether the number of correctly and incorrectly identified cases fitted a probability of 50%. For all comparisons, *P *< 0.05 was considered statistically significant (two-tailed). If the 95% CI of the difference did not include the value 0, the difference was statistically significant at α = 0.05. Systat 12, SigmaPlot 11 and SigmaStat 3.11 for Windows software (Systat Inc., Richmond, CA, USA) were used for the statistical analysis.

## Results

**Figure 8:**
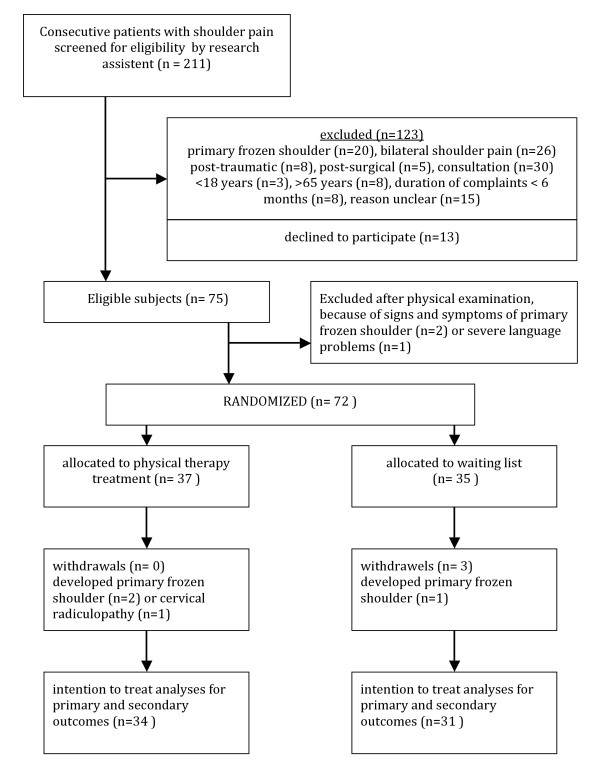
**Schematic showing patient participation**.

Between September 2007 and September 2009, 72 patients were randomly assigned to either the intervention group or the control group. See Figure 8 for the schematic summary of patient participation and Table 1 for the patients' characteristics at baseline. At baseline, both groups were comparable with regard to all variables and had no statistically or clinically relevant differences, except for the number of muscles with latent MTrPs and the patients' level of education (Table 1).

**Table 1:** Characteristics of participants at baselinea

Parameter	Intervention (n = 34)	Control (n = 31)
Age, mean yr (SD; 95% CI)	42.8 (11.7; 38.7-46.9)	45.0 (13.2; 40.2-49.9)
Female, number (%)	21 (62)	23 (74)
Level of education^b^, number (%)		
Low	2 (6)	2 (7)
Intermediate	13 (38)	17 (55)
High	19 (56)	12 (38)
Right-handed, number (%)	33 (97)	29 (94)
Pain dominant side, number (%)	24 (70)	19 (61)
Duration of complaints, number (%)		
6-9 months	10 (29)	5 (16)
9-12 months	4 (12)	8 (26)
1-2 yr	8 (23)	6 (19)
2-5 yr	6 (18)	5 (16)
>5 yr	6 (18)	7 (23)
Episode, number (%)		
First	13 (38)	11 (35)
Second	8 (24)	8 (26)
Third or more	13 (38)	12 (39)
DASH-DLV, mean (SD; 95% CI)^c^	30.3 (16.6; 24.5-36.1)	30.8 (11.9; 26.5-35.2)
VAS-P1, mean (SD; 95% CI)^d^	31.9 (24.3; 21.9-41.9)	35.2 (25.7; 25.7-43.0)
VAS-P2, mean (SD; 95% CI)^d^	41.3 (19.7; 33.2-49.4)	43.4 (17.0; 37.2-50.0)
VAS-P3, mean (SD; 95% CI)^d^	54.9 (21.9; 45.8-63.9)	59.5 (18.2; 52.8-66.2)
BDI-II-DLV, mean (SD; 95% CI)^e^	6.3 (4.0; 4.9-7.8)	5.8 (8.2; 2.8-8.8)
RAND-36-DLV, mean (SD; 95% I)^f^		
Social functioning	78.7 (20.3; 71.6-85.8)	81.1 (18.5; 74.3-87.8)
Limitations due to physical problems	47.7 (43.0; 32.5-63.0)	49.5 (37.2; 35.8-63.1)
Vitality	59.3 (17.0; 53.3-65.1)	62.6 (17.9; 56.0-69.1)
Bodily pain	51.6 (16.0; 45.7-57.6)	52.7 (12.3; 48.2-57.2)
General health perception	52.9 (8.5; 50.0-55.9)	56.6 (7.0; 54.1-59.2)
PROM, mean (SD; 95% CI)^g^	28.4 (34.8; 16.1-40.7)	39.0 (34.9; 26.2-51.8)
Muscles with MTrPs, mean (95% CI)^h^		
Active MTrPs	7.4 (3.6; 6.1-8.7)	6.1 (3.5; 4.8-7.4 )
Latent MTrPs	4.2 (2.7; 3.2-5.1)	5.9 (3.0; 4.8-7.0)

^a^SD, standard deviation; 95% CI, 95% confidence interval; MTrPs, myofascial trigger points. ^b^High education (university and higher vocational school), medium education (middle vocational school and higher or middle general secondary school) and low education (lower vocational school, lower general secondary school, primary school or no education). ^c^Higher Disabilities of the Arm, Shoulder and Hand outcome measure, Dutch-language version (DASH-DLV) scores indicate more disability, with a maximum of 100 (range, 0-100). ^d^Higher scores on the Visual Analogue Scale for Pain (VAS-P) indicate more pain, with a maximum of 100 (range, 0-100). VAS-P1, current pain score; VAS-P2, average pain score for the past 7 days; VAS-P3, most severe pain score for the past 7 days. ^e^Higher scores on the Beck Depression Inventory, 2nd edition, Dutch-language version (BDI-II-DLV) indicate more symptoms of depression (range, 0-63). ^f^Only the subscales of the nine subscales of the RAND Medical Outcomes Study 36-Item Short Form Health Survey, Dutch-language version (RAND-36-DLV) that differ significantly from a normal Dutch population are presented here [89]. Higher scores indicate a better quality of life (range, 0-100). ^g^A positive number (degrees) of the Passive Range of Motion (PROM) mean score indicates impairment of the PROM of the affected shoulder. ^h^Number of muscles with active, respectively latent MTrPs (range, 0-17 muscles).

### Primary outcome measure: DASH questionnaire

**Figure 9:**
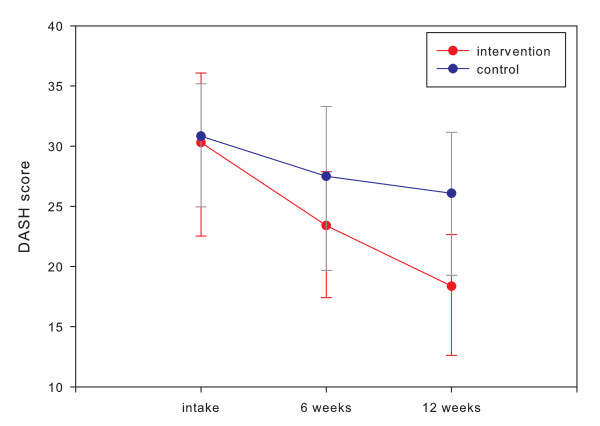
**The mean Disability of Arm, Shoulder, and Hand outcome measure (DASH) scores (error bars represent 95% confidence intervals) at intake, after 6 weeks and after 12 weeks for the intervention group (n = 34) and the control group (n = 31)**.

The difference between the intervention group and the control group was not significant after 6 weeks (4.1; 95% CI, -2.8 to 11.1) and was significant after 12 weeks (7.7; 95% CI, 1.2 to 14.2). The graphic presentation of the mean DASH questionnaire scores at intake and after 6 and 12 weeks is shown in Figure 9.

**Figure 10:**
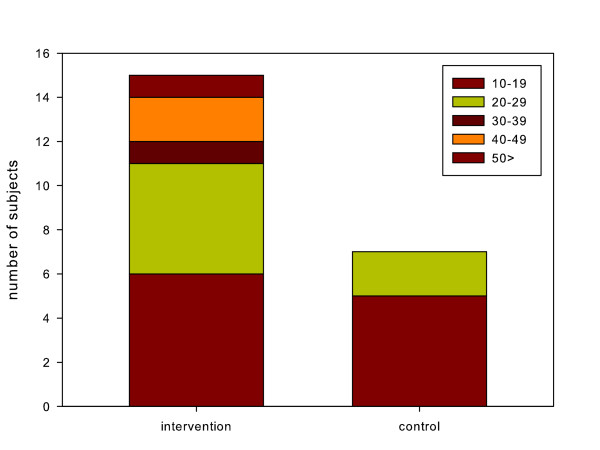
**The number of patients who improved by more than 10 points (minimal clinically important difference) on the DASH outcome measure after 12 weeks for the intervention group (n = 34) and the control group (n = 31)**.

Seventeen patients (50%) in the intervention group and seven (22%) in the control group improved by more than 10 points (MCID) on the DASH outcome measurement (RR, 2.3; 95% CI, 1.1 to 4.7) (Figure 10). The effect size (Cohen's *d*) was 0.60 (Table 2).

**Table 2:** Primary and secondary outcomes in the intervention group and the control group after 6 and 12 weeks^a^

Outcome	Intervention (n = 34)	Control (n = 31)	Mean difference (95% CI)	* P *value	Effect size (Cohen's *d*)
DASH, mean (SD)^b^					
Baseline	30.3 (16.6)	30.8 (11.9)	0.5 (-6.7-7.7)	NS	
After 6 wk	23.4 (12.6)	27.5 (15.5)	4.1 (-2.8-11.1)	NS	
After 12 wk	18.4 (12.3)	26.1 (13.8)	7.7 (1.2-14.2)	<0.05	0.60
VAS-P1, mean (SD)^c^					
Baseline	31.9 (24.3)	35.2 (25.7)	3.3 (-9.1-15.7)	NS	
After 6 wk	29.0 (18.4)	37.8 (17.9)	8.8 (-0.2-17.8)	NS	
After 12 wk	17.2 (19.5)	31.0 (21.0)	13.8 (2.6-25.0)	<0.05	0.69
VAS-P2, mean (SD)^c^					
Baseline	41.3 (19.7)	43.4 (17.0)	2.0 (-7.1-11.1)	NS	
After 6 wk	32.9 (19.3)	40.0 (20.7)	6.7 (-3.6-17.0)	NS	
After 12 weeks	22.5 (16.4)	33.2 (23.3)	10.2 (0.7-19.7)	<0.05	0.54
VAS-P3, mean (SD)^c^					
Baseline	54.9 (21.9)	59.5 (18.2)	4.6 (-14.6-5.4)	NS	
After 6 wk	41.0 (25.1)	56.6 (28.3)	15.6 (2.3-28.8)	<0.05	
After 12 wk	34.0 (21.9)	47.8 (27.3)	13.8 (0.8-28.4)	<0.05	0.57
GPE, number of patients (%)					RR (95% CI)
Improved					
After 6 wk	16/33 (49%)	5/30 (17%)		<0.05	2.9 (1.2-7.0)
After 12 wk	18/33 (55%)	4/28 (14%)		<0.05	3.8 (1.5-10.0)
Number of muscles with active trigger points, mean (SD)					
Baseline	7.4 (3.7)	6.1 (3.5)	1.3 (-0.5-3.1)	NS	
After 6 wk	6.2 (3.5)	6.8 (3.6)	0.6 (-1.2-2.4)	NS	
After 12 wk	4.8 (3.0)	7.5 (3.2)	2.7 (1.2-4.2)	<0.05	0.89
Number of muscles with latent trigger points, mean (SD)					
Baseline	4.2 (2.7)	5.9 (3.0)	1.7 (-0.3-3.1)	<0.05	
After 6 wk	3.8 (2.1)	4.8 (2.8)	1.0 (-2.3-0.2)	NS	
After 12 wk	4.7 (2.3)	4.4 (2.3)	0.4 (-0.7-1.5)	NS	0.13

^a^95% CI, 95% confidence interval; SD, standard deviation; ES, effect size; NS, not significant; RR, relative ratio; GPE, global perceived effect. ^b^Higher scores on the Disabilities of the Arm, Shoulder and Hand outcome measure, Dutch-language version (DASH-DLV) and more disability, with a maximum of 100 (range, 0-100). ^c^Higher scores on the Visual Analogue Scale for Pain (VAS-P) indicate more pain, with a maximum of 100 (range, 0-100). VAS-P1 represents the current pain score, VAS-P2 represents the average pain score for the past 7 days, and VAS-P3 represents the most severe pain score for the past 7 days.

The multiple linear regression analysis with the baseline score as a covariate demonstrated a significantly higher DASH questionnaire score at 12 weeks of 7.447 (95% CI, 2.14 to 12.75) in the intervention group compared with the control group. Adjustment for the covariates had no influence on this result.

### Secondary outcomes

#### VAS-P1, VAS-P2 and VAS-P3

The intervention group showed, on average, significantly lower scores on all VAS-P scales compared with the control group after 12 weeks: VAS-P1 (13.8; 95% CI, 2.6 to 25.0), VAS-P2 (10.2; 95% CI, 0.7 to 19.7) and VAS-P3 (13.8; 95% CI, 0.8 to 28.4). The differences after 6 weeks were not significant except for VAS-P3 (15.6; 95% CI, 2.3 to 28.8). The difference between baseline and after 12 weeks in the intervention group reached the MCID for all three VAS-P scales, while changes in the control group did not reach the MCID. The effect sizes on the three VAS-P scales varied from 0.5 to 0.7 (Table 2).

#### GPE

After 6 weeks, improvement was reported by 16 (49%) of 33 patients in the intervention group versus 5 (17%) of 30 patients in the control group (RR, 2.9; 95% CI, 1.2 to 7.0). After 12 weeks, 18 (55%) of 33 patients in the intervention group self-reported to be improved versus 4 (14%) of 28 patients in the control group (RR, 3.8; 95% CI, 1.46 to10.0) (Table 2).

### Number of muscles with trigger points

The number of muscles with active MTrPs was significantly lower in the intervention group than in the control group after 12 weeks (mean difference, 2.7; 95% CI, 1.2 to 4.2). The change in the number of muscles with latent MTrPs was nonsignificant compared with the control group (mean difference, 0.4; 95% CI, -0.7 to 1.5) (Table 2). The effect size (Cohen's *d*) for active MTrPs after 12 weeks was 0.89, a large effect, and for latent MTrPs it was 0.13.

### Correlation between the number of muscles with active MTrPs and the DASH questionnaire outcome at 12 weeks

The number of shoulder muscles with active MTrPs was positively correlated with the DASH questionnaire outcome at 12 weeks (*r *= 0.49, regression coefficient = 2.13, *P *= 0.000, ANOVA *P *= 9.6; *P *= 0.000, when corrected for muscles with active MTrPs at intake). This implies that the number of muscles with active MTrPs was associated with 24% of the variation in DASH questionnaire outcome. Two cases were identified as significant outliers during the multiple linear regression analysis (both in the intervention group) and were removed before further analysis.

#### PROM

The PROM difference between the groups did not change significantly during the measurements at 6 weeks (mean difference, 8.8; *t *= 1.14; *P *> 0.05) and at 12 weeks (mean difference, 8.2; *t *= 1.19; *P *> 0.05).

### Evaluation of blinding

After 6 weeks, the observers identified the treatment allocation correctly in 62% of the patients (χ^2 ^= 4.70; *P *= 0.03) and after 12 weeks in 71% of the patients (χ^2 ^= 13.86; *P *= 0.00) after completing the physical examination and MTrP count.

### Cointerventions

We checked whether the participants in either group had received interventions other than those described in the treatment protocol. During the first 6 weeks of the study, one individual in each group received an injection administered by a general practitioner. After 6 weeks, no cointerventions were reported in either group.

## Discussion

### Summary of main findings

This single-blinded RCT evaluated the effectiveness of a 12-week comprehensive MTrP physical therapy treatment program in patients with chronic, nontraumatic, unilateral shoulder pain when compared with a wait-and-see strategy. After 12 weeks, the intervention group showed statistically as well as clinically significant differences compared with the control group on the primary and secondary outcome measures. The effect sizes were considered to be medium and consistent with the hypothesized effect size. The number of shoulder muscles with active MTrPs was significantly lower in the intervention group than in the control group, supporting the assumed biomedical mechanism underlying MTrP therapy.

### Explaining the results and comparing them with those of other studies

To our knowledge, this is the first study of the effectiveness of a comprehensive MTrP therapy program in patients with shoulder pain. The difference [74] of the DASH questionnaire scores between groups was smaller than the MCID. However, the mean of the baseline DASH questionnaire score was smaller than expected on the basis of results from other studies [56,75,76]. With a smaller mean value, observation of great differences between baseline and follow-up at 12 weeks is less likely. However, the effect size was 0.6, which is considered to be a medium effect that is clinically relevant. The number of patients who improved by more than 10 points in this study is a clinically relevant result. Furthermore, many more patients in the intervention group than in the control group reported improvement according to the GPE scale.

Researchers in previous trials have investigated various types of physical, manual and exercise therapy. The treatments in these studies included interventions showing similarities to components of the treatment program of this study, but were not aimed specifically at treating MTrPs. For example, exercise therapy or manual therapy interventions included soft tissue massage and muscle-stretching exercises, which generally are performed for anterior and posterior muscle tightness [74,77-79]. These interventions may have an unintentional effect on MTrPs in shoulder muscles because MTrPs seem to be prevalent in patients with shoulder pain, which may have contributed to the results of other studies [[25]; Bron et al, unpublished work]. However, because these studies did not focus on MTrPs, there is no direct evidence that these interventions did have or did not have an effect on MTrPs.

Recently, Hains *et al*. [52] published the first report on the effectiveness of ischemic compression therapy of MTrPs in shoulder muscles in patients with chronic shoulder conditions compared with sham compression. The intervention group underwent 15 sessions of therapy (comprising 15-second compression of MTrPs in up to four muscles, including the supraspinatus, infraspinatus, deltoid and biceps) three times weekly without any other therapeutic measures. The control group received sham therapy (15 seconds of compression of MTrPs in shoulder muscles, which is considered irrelevant for shoulder pain). The intervention group showed a significant improvement on the Shoulder Pain and Dysfunction Index compared with the sham group [52]. Hains *et al*. did not report any change in the number of MTrPs in the shoulder muscles or in the number of shoulder muscles with MTrPs. The current study has shown that a decrease in the number of shoulder muscles with active MTrPs is correlated with better outcome. While the number of muscles with active MTrPs decreased in the intervention group, the number of muscles with latent MTrPs tended to increase slightly. One explanation might be that the state of MTrPs is more or less dynamic and that changes from active to latent and vice versa occur, depending on the degree of irritability [80].

One of the clinical features of active MTrPs is spontaneous pain at rest or during activity which is felt at a site distant from the MTrP side and, by definition, has to be recognized by the patient as familiar pain. According to Mense, "The current concept of the referral of muscle pain is based on the observation that the efficacy of synaptic connections of central dorsal horn neurons can change, particularly under the influence of a nociceptive input. The important point is that ineffective synaptic connections can become effective under pathological circumstances. This means that a neuron can acquire new receptive fields in the presence of nociceptive input" [[81], page 350]. This process is called central sensitization. By expanding receptive fields, non-nociceptive input originating from a location other than the originally painful location may be perceived as painful. In patients with shoulder pain, MTrPs in the infraspinatus, supraspinatus, teres minor or subscapularis muscle, for example, may cause local and referred pain, which can be felt deep within the shoulder. In other words, MTrPs may mimic pain interpreted as pain arising from subacromial bursitis, tendonitis or tendonopathy, which may explain why treatment of inflammation is so often ineffective.

Furthermore, MTrPs can cause particular motor effects as well. MTrPs can lead to muscle weakness of the involved painful muscle and reorganization of motor activation patterns. Restricted range of motion may be observed secondary to a contracted taut band [80,82,83]. A changed motor activation pattern has often been reported in the shoulder pain literature [84]. Since MTrPs can alter such patterns, MTrP inactivation should be considered prior to any form of muscle-strengthening exercises. When muscle weakness persists, it may alter a patient's shoulder kinematics and eventually cause humeral head migration, rotator cuff degeneration and formation of bony spurs in the subacromial space. Early recognition and treatment of MTrPs may prevent the development of chronic shoulder pain and early degeneration.

As we did not examine the effects of single components of the intervention, we cannot conclude whether a single component or a combination of components contributed more to the treatment effect than other components. Others have examined the effect of single ischemic compression or a combination of ischemic compression and stretching and concluded that both interventions had positive effects on patients' recovery [44]. The management of MTrPs is not restricted to MTrP inactivation, but it requires correction of perpetuating factors that are clinically apparent but not yet necessarily scientifically established [26,41,43]. Further research is needed to clarify the importance of perpetuating factors, such as mechanical factors, in patients with shoulder pain [85].

### Limitations of the study

The power analysis indicated that 104 patients were needed for this clinical trial. Partly because of an overestimation of the number of eligible patients and partly because of the unwillingness of patients to enter the trial, the study was completed with a sample size smaller than 104. This study took 2 years to complete, which is 1 year longer than originally planned. However, the results are significant and clinically relevant, although the study population was smaller than the initially calculated sample size. A greater sample size would be unlikely to have altered the direction of the results.

The participants in the intervention group had a higher level of education than those in the control group. Awareness of educational levels is important, as it may affect patients' motivation and compliance [86,87], but adding the level of education as a covariate in multiple linear regression analysis did not alter the results.

Evaluation of the blinding of the independent observers, who performed the physical examination and the counting of MTrPs, revealed that after 12 weeks the observers were able to identify to which group a patient belonged. It is likely that the changes in physical findings and the decrease in the number of MTrPs improved the observers' accuracy of group identification. Since the blinding influenced only the observer who performed the MTrP identification, this finding had no effect on the reliability of the other outcome scores.

The patients in the control group were instructed to maintain self-management of their shoulder pain and to report any management deviation. While this factor may pose a potential threat to the comparability of the groups, no significant changes were reported. As all patients had chronic shoulder pain and likely had explored various self-management strategies before entering into the study, we did not anticipate that they would change their self-management strategies during the study period.

Although the observers did not intend to give some good advice during the physical examination, they may have unintentionally instructed the patients to avoid provocative activities. When the patients in the control group followed the instructions and acted more carefully during their daily lives, their symptoms may have been reduced while they still had MTrPs. This may explain the improvement in the control group.

### Implications for research and clinical practice

This study shows that patients with chronic, unilateral, nontraumatic shoulder pain had better outcomes after treatment for MTrPs than did patients without treatment, and this outcome was correlated with a decrease in the number of muscles with active MTrPs.

Treatment of MTrPs can be considered a promising approach for the treatment of patients with shoulder pain. Future clinical trials should be directed toward establishing the effectiveness of MTrP treatment in patients with varying underlying pathologies of the shoulder and in a wider context than a specialized physical therapy practice. It would be worthwhile to identify predictors of successful MTrP treatment and to investigate whether MTrP treatment is more successful when combined with supportive interventions such as exercise and manual therapy. Observational follow-up studies are needed to investigate the long-term effects of treatment of MTrPs in patients with chronic shoulder pain. Given the high number of patients with shoulder pain, this will require substantial effort and financial investment. Studies on the cost-effectiveness of treatment of patients with MTrPs in the shoulder muscles are therefore needed.

## Conclusions

Participants in the intervention group had better outcomes on all outcome measures after 12 weeks of a comprehensive MTrP treatment program than did those on the waiting list. Clinically relevant improvements were achieved in 55% of the patients with shoulder pain, and the number of muscles with active MTrPs was significantly decreased.

## Competing interests

The authors declare that they have no competing interests.

## Authors' contributions

All authors read, edited and approved the final manuscript. CB is the lead investigator of the study and developed its design, carried out data acquisition, data analysis and interpretations, and was the primary author of the manuscript. MW and RO supervised the study and helped to prepare the manuscript. JD, BS and AdG provided intellectual contributions to the manuscript.

## Appendix. List of muscles examined for myofascial trigger points

Upper trapezius muscle

Middle trapezius muscle

Lower trapezius muscle

Infraspinatus muscle

Supraspinatus muscle

Subscapularis muscle

Teres minor muscle

Teres major muscle

Anterior deltoid muscle

Middle deltoid muscle

Posterior deltoid muscle

Pectoralis major muscle

Pectoralis minor muscle

Biceps brachii muscle

Triceps brachii muscle

## Pre-publication history

The pre-publication history for this paper can be accessed here:

http://www.biomedcentral.com/1741-7015/9/8/prepub
